# Manipulation of Human Verticality Using High-Definition Transcranial Direct Current Stimulation

**DOI:** 10.3389/fneur.2018.00825

**Published:** 2018-10-22

**Authors:** Taiza E. G. Santos, Diandra B. Favoretto, Iman Ghodratti Toostani, Diego C. Nascimento, Brunna P. Rimoli, Eduardo Bergonzoni, Tenysson Will Lemos, Dennis Q. Truong, Alexandre C. B. Delbem, Bahador Makkiabadi, Renato Moraes, Francisco Louzada, Marom Bikson, Joao P. Leite, Dylan J. Edwards

**Affiliations:** ^1^Department of Neuroscience and Behavioral Sciences, Ribeirao Preto Medical School University of Sao Paulo, Ribeirão Preto, Brazil; ^2^Neurocognitive Engineering Laboratory, Center of Engineering Applied to Health University of São Paulo, São Carlos, Brazil; ^3^Reconfigurable Computing Laboratory, Institute of Mathematics and Computer Sciences University of São Paulo, São Carlos, Brazil; ^4^Department of Applied Mathematics and Statistics, Institute of Mathematics and Computer Sciences University of São Paulo, São Carlos, Brazil; ^5^School of Physical Education and Sport of Ribeirao Preto University of São Paulo, Ribeirao Preto, Brazil; ^6^Neural Engineering Laboratory, Department of Biomedical Engineering, The City College of New York, City University of New York New York, NY, United States; ^7^Department of Medical Physics and Biomedical Engineering, School of Medicine Tehran University of Medical Sciences (TUMS), Tehran, Iran; ^8^Research Center for Biomedical Technology and Robotics (RCBTR), Institute of Advanced Medical Technologies (IAMT) Tehran University of Medical Sciences, Tehran, Iran; ^9^Moss Rehabilitation Research Institute Elkins Park, PA, United States; ^10^School of Medical and Health Sciences, Edith Cowan University Joondalup, WA, Australia

**Keywords:** high-definition transcranial direct current stimulation, temporo-parietal junction, verticality, postural control, electroencephalography

## Abstract

**Background:** Using conventional tDCS over the temporo-parietal junction (TPJ) we previously reported that it is possible to manipulate subjective visual vertical (SVV) and postural control. We also demonstrated that high-definition tDCS (HD-tDCS) can achieve substantially greater cortical stimulation focality than conventional tDCS. However, it is critical to establish dose-response effects using well-defined protocols with relevance to clinically meaningful applications.

**Objective:** To conduct three pilot studies investigating polarity and intensity-dependent effects of HD-tDCS over the right TPJ on behavioral and physiological outcome measures in healthy subjects. We additionally aimed to establish the feasibility, safety, and tolerability of this stimulation protocol.

**Methods:** We designed three separate randomized, double-blind, crossover phase I clinical trials in different cohorts of healthy adults using the same stimulation protocol. The primary outcome measure for trial 1 was SVV; trial 2, weight-bearing asymmetry (WBA); and trial 3, electroencephalography power spectral density (EEG-PSD). The HD-tDCS montage comprised a single central, and 3 surround electrodes (HD-tDCS3x1) over the right TPJ. For each study, we tested 3x2 min HD-tDCS3x1 at 1, 2 and 3 mA; with anode center, cathode center, or sham stimulation, in random order across days.

**Results:** We found significant SVV deviation relative to baseline, specific to the cathode center condition, with consistent direction and increasing with stimulation intensity. We further showed significant WBA with direction governed by stimulation polarity (cathode center, left asymmetry; anode center, right asymmetry). EEG-PSD in the gamma band was significantly increased at 3 mA under the cathode.

**Conclusions:** The present series of studies provide converging evidence for focal neuromodulation that can modify physiology and have behavioral consequences with clinical potential.

## Introduction

After nearly two decades of experimentation with transcranial direct current stimulation (tDCS), including hundreds of registered trials, over 1,000 published manuscripts and use extending outside of the laboratory (1), few tDCS protocols have achieved robust scientific acceptance, and evidence of dose-response effects of tDCS are ambiguous and limited (2–4).

It has been shown that conventional tDCS using a bipolar pad-electrode montage results in a dispersed area of stimulation and non-linear effects, where increasing intensity does not necessarily increase efficacy (2, 5). An alternative approach uses circular small diameter gel-based electrodes, with tight configuration, called high-definition tDCS (HD-tDCS) and results in a more focused stimulation area (6, 7) and is considered promising for neurorehabilitation applications. We recently demonstrated using both modeling and physiological data that a HD-tDCS montage can achieve substantially greater cortical stimulation focality than conventional electrode tDCS (7).

The target for neuromodulation techniques depends on the neural network node for which it is reasoned that excitability modulation will have functional relevance. The temporo-parietal junction (TPJ) may be a rational target region in post-stroke postural imbalance as it is a critical hub for multisensory integration and processing (8, 9). Furthermore, the TPJ plays a pivotal role in human perception for verticality and postural control (9–13).

After stroke, vertical misperception and postural imbalance are frequent and negatively impact functional recovery (14–17). Targeting the TPJ, we recently showed that it is possible to manipulate perception of verticality (18) and postural control (19) in a polarity dependent manner during and after conventional tDCS over TPJ bilaterally (bipolar-balanced montage) (20). These studies provided support for restoration of visual vertical misperception and improved postural imbalance after stroke using tDCS over TPJ (19). There is a critical need, however, to step back and establish dose-response effects in the intact nervous system using well-defined protocols with relevance to clinically meaningful applications.

Here we present converging evidence for manipulation of human verticality using a novel HD-tDCS montage from three separate randomized, double-blind, crossover clinical trials in healthy adults, spanning behavioral and physiological outcomes.

We selected an HD-tDCS montage based on modeling data (see **Figure 2**), where the cortical electric field would be largely confined within the boundary of the surround electrodes and would be centered over the TPJ. While 4 × 1 concentric HD-tDCS configurations (4 surrounding cathodes and a center anode or vice versa) have been previously modeled and experimentally tested to fit these constraints (7), asymmetric 3 × 1 HD-tDCS configurations have not. Previous models, however, suggest similar focality and intensity distributions between 4 × 1 and 3 × 1 configurations (21). Due to ergonomic constraints around the ear and to avoid potential confounding stimulation sites, a surround electrode was omitted from a typical 4 × 1 configuration leaving 3 surrounding electrodes and one center electrode. We tested stimulation intensities of 1, 2, and 3 mA.

The visual vertical trial examined a polarity and intensity-dependent shift in perception of visual vertical following HD-tDCS over the right TPJ. Based on our preliminary studies, we hypothesized that judgment error in perception of vertical would progressively increase with increasing stimulus intensity, and that the intensity dependent effects would be observed only for cathode center, producing specific leftwards tilt, whereas sham and anode center conditions would produce no effect.

The postural control trial aimed to demonstrate polarity and intensity-dependent postural asymmetry following HD-tDCS over the right TPJ. Stroke patients that can stand still frequently present with greater loading on the ipsilesional foot associated with contralesional visual vertical tilt (22). We hypothesized a rightward weight-bearing asymmetry (WBA) after cathode center and leftward after the anode center condition. We expected to observe intensity dependent effects for both anodal and cathodal polarity.

The electroencephalography (EEG) trial investigated a polarity and intensity-dependent shift in EEG power spectral density (PSD) following HD-tDCS over the right TPJ, and whether this would be anatomically confined to the targeted TPJ, and whether changes might occur remotely in the contralateral cortical homolog. Based on prior tDCS-EEG studies (23), together with polarity-dependent changes in excitability probed using the motor evoked potential (24), we hypothesized that the magnitude of the local gamma band EEG-PSD change after HD-tDCS would increase with intensity increase; and that the intensity dependent effects on EEG-PSD change would be polarity dependent.

After HD-tDCS over the right TPJ, we found intensity and polarity-dependent effects on visual vertical perception, intensity-dependent effects on gamma band EEG-PSD and polarity-dependent effects on postural asymmetry with only few mild adverse effects. The present findings show for the first time, feasibility, safety and efficacy for HD-tDCS_3 × 1_ over the TPJ multimodal cortex, setting the stage for use of the HD-tDCS_3 × 1_ montage for other cortical targets.

## Materials and methods

### Overview

This study was conducted according to the Helsinki Declaration requirements for human investigation, and was approved by the local ethics committee. All participants provided written informed consent.

In the subsequent sections, we will present the common features of the 3 clinical trials (stimulation montage and intensity) followed by the details of the outcome measures of each trial: visual vertical, postural control and EEG.

### Participants

Each clinical trial included distinctive sample population naïve and blind to the HD-tDCS_3 × 1_ approach and the study purpose.

Study candidates were healthy subjects with no evidence of brain, vestibular or orthopedic dysfunction, aged from 18 to 50 years old, with normal or corrected-to-normal vision. Oculomotor tests, the head shake and head thrust test, were performed to guarantee the exclusion of vestibular deficits.

### Intervention

HD-tDCS was applied using 3 × 1 ring electrode configuration (HD-tDCS_3 × 1_) in all clinical trials. The center electrode was placed in the circumcenter of a triangle with vertices on C4, T4, P4 over the right hemisphere. The 3 surround electrodes were placed over P4, C4, and T8. Direct current (DC) was generated by a 1 × 1 DC stimulator and then split into the 3 high-density Ag/AgCl sintered ring electrodes (Soterix Medical®, NY-USA).

**Figure 1:**
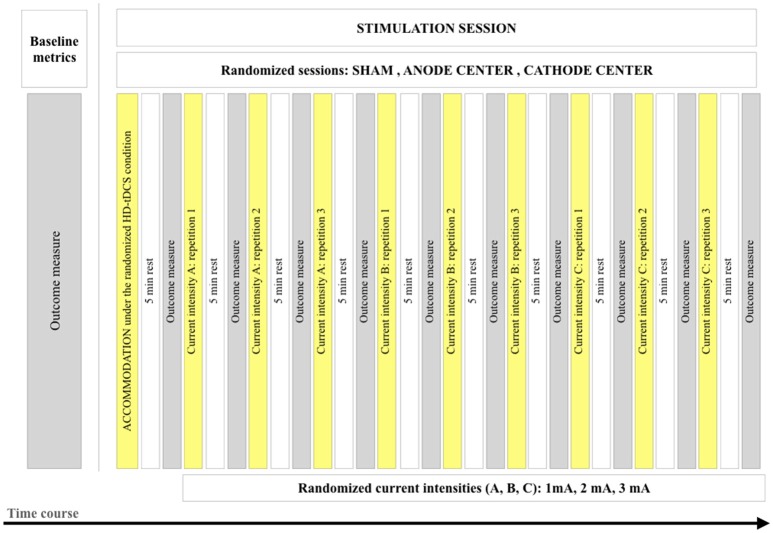
Illustration of the stimulation protocol and time course of the study.

In a seated position, participants received 3 HD-tDCS stimulation conditions (anode center, cathode center, and sham) on 3 different days, with an interval of at least 24 h. The sham condition consisted of the same positioning of the electrodes, but current followed a ramp-up of 30 s and a subsequent ramp-down of 30 s. Before the beginning of stimulation, baseline data were collected. The first stimulation procedure was designed to desensitize the participants in relation to the current and termed *HD-tDCS*_3__×*1*_ *accommodation* protocol applied in every session (Supplementary Material 1). It consisted of 3 repetitions of 5 s of stimulation at current intensities of 1, 2, and 3 mA, with ramp-up and ramp-down of 2 s/mA and intervals of 5 s, performed by the investigator that followed a video to guarantee reproducibility. The HD-tDCS_3 × 1_ stimulation protocol consisted of 3 blocks at each current intensity (1, 2, and 3 mA) with random order, and an inter-stimulus interval between blocks of 5 min. The stimulation duration of each block was 2 min. The total stimulation duration of the protocol was 19.8 min (accommodation and stimulation protocols), excluding the ramp periods. Figure 1 illustrates the stimulation protocol and time course of the study.

#### Dose calculation of each stimulation session

The dose of each tDCS session was defined by electrode montage (skin contact area/size and position of all electrodes), stimulation intensity and duration^2^. The stimulation charge (current intensity × duration) of the present protocol was the following:

$$\begin{array}{l} \text{Accommodation protocol}=\;3\;\mathrm{bl}ocks\;of\;5s\;of\;stimulation\\ \;at\;current\;intensities\;of\;1,\;2,\;and\;3\;mA\\ \text{Accommodation protocol}=3^{*}\left[\left(5^{*}1\right)+\left(5^{*}2\right)+\left(5^{*}3\right)\right]=90\;\text{m}\text{C}\\ \text{Stimulation protocol }=9\;blocks\;of\;2\min of\;stimulation\\ \text{Stimulation protocol }=3^{*}\left[\left(1^{*}120\right)+\left(2^{*}120\right)+\left(3^{*}120\right)\right]\\ \;=2160\;\text{m}\text{C}\\ \begin{array}{c} \text{Total charge of the}\\ \text{active session } \end{array}=\text{Accommodation}+\text{Stimulation}\\ \;=2250\;\text{m}\text{C} \end{array}$$

Charge is a summary metric that depends on several parameters, but is insensitive to other potentially significant parameters such as polarity (25), and is reported here for future protocol reproducibility (7). A computational model of the induced current flow was also developed to estimate penetration in the region of interest.

#### Modeling of the HD-tDCS induced current flow

Brain stimulation as represented by cortical electric field and the current density was predicted during tDCS by modeling electrostatic physics with Finite Element (FE) models. Three-dimensional head models consisting of tissues with varying material properties (conductivities) were created using sample anatomical MRI data. MRI scans (1 mm^3^ resolution) were previously segmented into 7 materials of varying conductivity (S/m): skin (0.465), fat (0.025), bone (0.01), cerebrospinal fluid or CSF (1.6), gray matter (0.276), white matter (0.126), and air (10–15). Circular electrodes (1 cm radius, 5.99e7 S/m) with a conductive gel layer (4 mm thickness, 1.4 S/m) were centered over the TPJ as in the experimental protocol. Surrounding electrodes were 5 cm from the TPJ and spaced 90 degrees apart in the furthest location from the mastoid. An adaptive volumetric mesh was generated using ScanIP (Simpleware, Exeter, UK). Volume conductor physics were applied in an FE package (COMSOL, Burlington, MA) and the voltage was solved for using the following boundary conditions: 1 mA inward current on the anode surface, ground on the cathodes, and insulation on the remaining exposed surfaces. The FE solution was then scaled to additional stimulation intensities of 2 and 3 mA. Cortical electric field magnitude was calculated to represent stimulation.

### Safety and tolerability

After each application of HDtDCS_3 × 1_, the assessor asked the participants about the degree of discomfort using visual analog scale from zero to 10. The participants were also instructed to report any adverse effects related to the study protocol after each session.

#### Stopping rule

The stopping rule was determined before the start of the experiments, where subjects would discontinue the study (all 3 trials) if any serious adverse effects related to the stimulation protocol occurred. Specific caution was given to the occurrence of a seizure or scalp burn at the site of the electrodes.

### Randomization

The randomization sequence was built using SAS/STAT Software (Statistical Analysis System, version 9.4). Block randomization with different block sizes was used for HD-tDCS_3 × 1_ condition (anode center, cathode center and sham), and current intensity (1, 2, and 3 mA). Simple balanced randomization within a single randomization block of four was used to determine the sham condition (anode center or cathode center).

### Blinding and allocation concealment

Participants, outcome assessors, and statisticians were blinded to the intervention. A third investigator applied the HD-tDCS_3 × 1_ and held the randomization sequence and allocation for each participant.

### Outcome measures

#### Visual vertical trial

**Figure 2:**
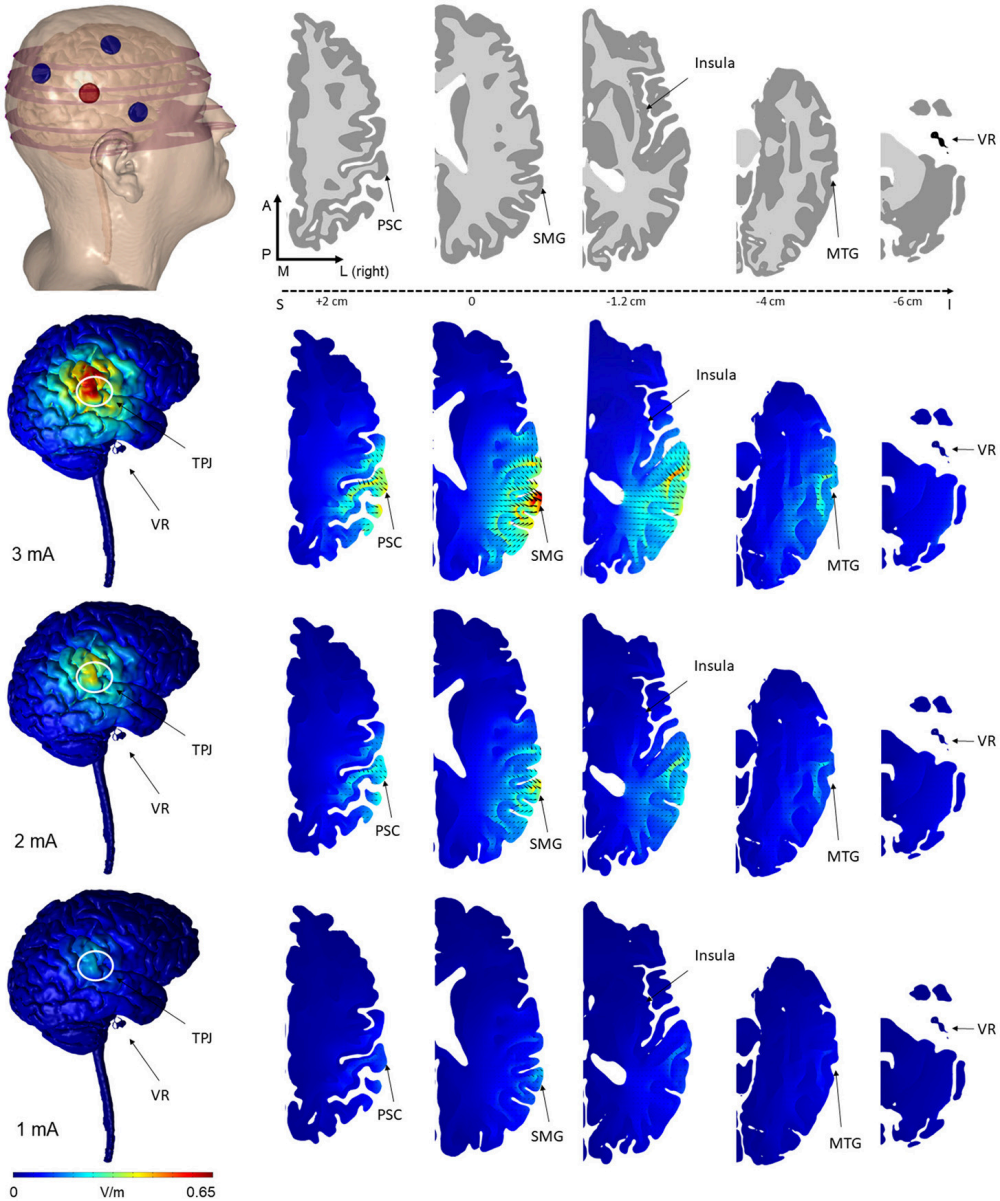
Finite element models of tDCS using the novel 3 × 1 HD-tDCS electrode montage over TPJ predicted the induced electric field on the brain. The 3 cathode−1 anode (or conversely 3 anode−1 cathode) montage produced peak electric field concentrated under the center electrode at the TPJ. While the relative spatial distribution is unchanged between stimulation intensities, electric field magnitude increases linearly from 1 to 3 mA. Note that for all montages, little electric field is induced at the vestibular apparatus. Electric field magnitude at the TPJ and other regions of interest were predicted at 3 intensities (Rows: 3, 2, 1 mA) and plotted on the same scale for comparison. Current density direction within each slice, analogous to stimulation polarity, was predicted as cones directed into the cortical surface for anode-center stimulation. The model geometry (slice location within the head, electrode position, and neuroanatomy) are illustrated in the top row (VR, vestibular receptors; TPJ, Temporo-Parietal Junction; PSC, Primary Somatosensory Cortex; SMG, Supramarginal Gyrus; MTG, Middle Temporal Gyrus).

Subjective visual vertical (SVV) was assessed before and after the stimulation and was determined using the *bucket method* (Figure 2) (26). To perform the assessments, each participant remained seated upright in a chair with the back and feet supported, and with their trunk restrained by bands. Participants visualized a black line (10.5 cm long, 0.4 cm wide, at 25 cm distance) at the center of the bucket base, internally. On the exterior surface of the bucket base, a protractor was aligned perpendicular to the dark line inside with a pendulum suspended from the axis of bucket rotation; and was used to calibrate a digital inclinometer with a precision of 0.01° before each session. A positive sign indicated clockwise SVV tilt and a negative sign a counterclockwise SVV tilt. Participants were asked to look into the bucket while the examiner manually rotated it slowly in clockwise (+SVV) and counterclockwise (–SVV) directions. Subjects verbally reported when the line inside the bucket appeared upright. The examiner rotating the bucket and supervising the position of the participant's head was blinded to the inclinometer measurements. A second examiner registered the SVV results. All subjects practiced at least six times to exclude a learning effect and were instructed to make as many corrections as required to set the line at their perceived vertical. SVV assessment was repeated 4 times, 2 beginning with the upper edge of the line in the clockwise and 2 in the counterclockwise direction in random order and angulation. The results were expressed by the mean of the 4 SVV assessments in degrees.

#### Postural control trial

Posturography was assessed before and after stimulation to determine WBA. Vertical ground reaction force (VGRF) was collected for each foot independently from two separate force platforms (Bertec 4060, Bertec Corporation, Columbus, Ohio-USA, size 40 × 60 cm) placed side-by-side, one under each foot. The acquisition rate was at 100 Hz. VGRF represents the weight distribution, and was low-pass filtered at 10 Hz using a fourth-order, phase-corrected Butterworth filter.

Participants were instructed to adopt a spontaneous stance with eyes closed, barefoot, one foot on each of the two platforms (heels separated by 9 cm, toe out at 30°), and arms hanging freely beside the body. Participants were asked to stand still during each assessment of 2 min, and rest seated during the intervals according to stimulation protocol. Since the posturography duration was 2 min, the final interval that participants remained without HD-tDCS_3 × 1_ stimulation was 7 min. The use of two force platforms yields direct information about possible weight bearing asymmetry. The inter-limb VGRF symmetry ratio was calculated. The results were expressed as the percentage of the total body weight loading with positive values when the WBA occurred toward the right side and negative values when WBA occurred toward the left side.

#### EEG trial

Dense array EEG was assessed with HD-tDCS and was acquired using a 256-channel sensor net from Electrical Geodesics Inc. with a sampling frequency of 500 Hz. All channels were referenced to the vertex with electrical impedance reduced to below 50 KΩ before data collection. The EEG was recorded continuously before and after the stimulation, including ramping up and ramping down periods. After data collection, EEG signals were high-pass filtered at 0.1 Hz, and re-referenced to an average reference. Channels 164 was selected for pilot data analysis as the most proximal position to the center electrode of the HD-tDCS_3 × 1_. Channel 66 was also selected because is in the homologous position in relation to channel 164.

The signals were segmented into 5 s blocks and the trend of each segment was eliminated using non-linear trend estimation method, and then the mean value of each segment removed. Next, the high amplitude noise (i.e., motion artifact) was detected in each segment and the signal value at the moments with such high amplitude, was replaced with NaN (not a number). Each segment of signal was filtered with a band-pass filter (BPF) in Theta (4–8 Hz), Alpha (8–12 Hz), Beta (12–30 Hz), and Gamma (30–100 Hz) frequency bands. Power-spectral density (PSD) of the estimated signals was calculated using the Welch method. Line noise (60 Hz) was eliminated by a Band Stop Filter. The filters were either Butterworth order 4, or a sharper filter (2 sequential filter order 4). The power of each segment was calculated for each channel and was saved into columns of power matrices.

The primary outcome measures were the four frequency bands. For the primary outcomes, the time-points of assessment collected in relation to each block of stimulation were baseline and 5 minutes after stimulation (prior to the ramp up of the next stimulation block).

### Statistical analyses

A graphical investigation was made in order to analyze the data distribution. Since raw data did not achieve the assumptions of normality, linearity, and homoscedasticity, a non-parametric approach was used, comparing the medians provided from the interaction of the experimental factors. SVV, WBA, and EEG-PSD were considered as outcome variables and the analyses were conducted according to statistical guidelines for the analysis of crossover studies (27).

The comparison among medians was rank based. The Kruskal–Wallis test was used as the global test to assess the effect of current intensity for each HD-tDCS condition (anode center, cathode center, or sham), and the effect of HD-tDCS condition, for each current intensity (1, 2, or 3 mA). The Tukey *post-hoc* test was used to compare the interventions only when the Kruskal–Wallis revealed statistical significance (*p* < 0.05). Since the hypotheses were defined a priori and we used a global test across comparison treatments, no adjustments for multiple comparisons were performed (28). In all tests, a 5% level of significance was used (two-sided). Statistical analyses were performed using R Project for Statistical Computing. The descriptive results of the figures are presented as the difference from baseline.

## Results

### Modeling of the HD-tDCS induced current flow

**Figure 3:**
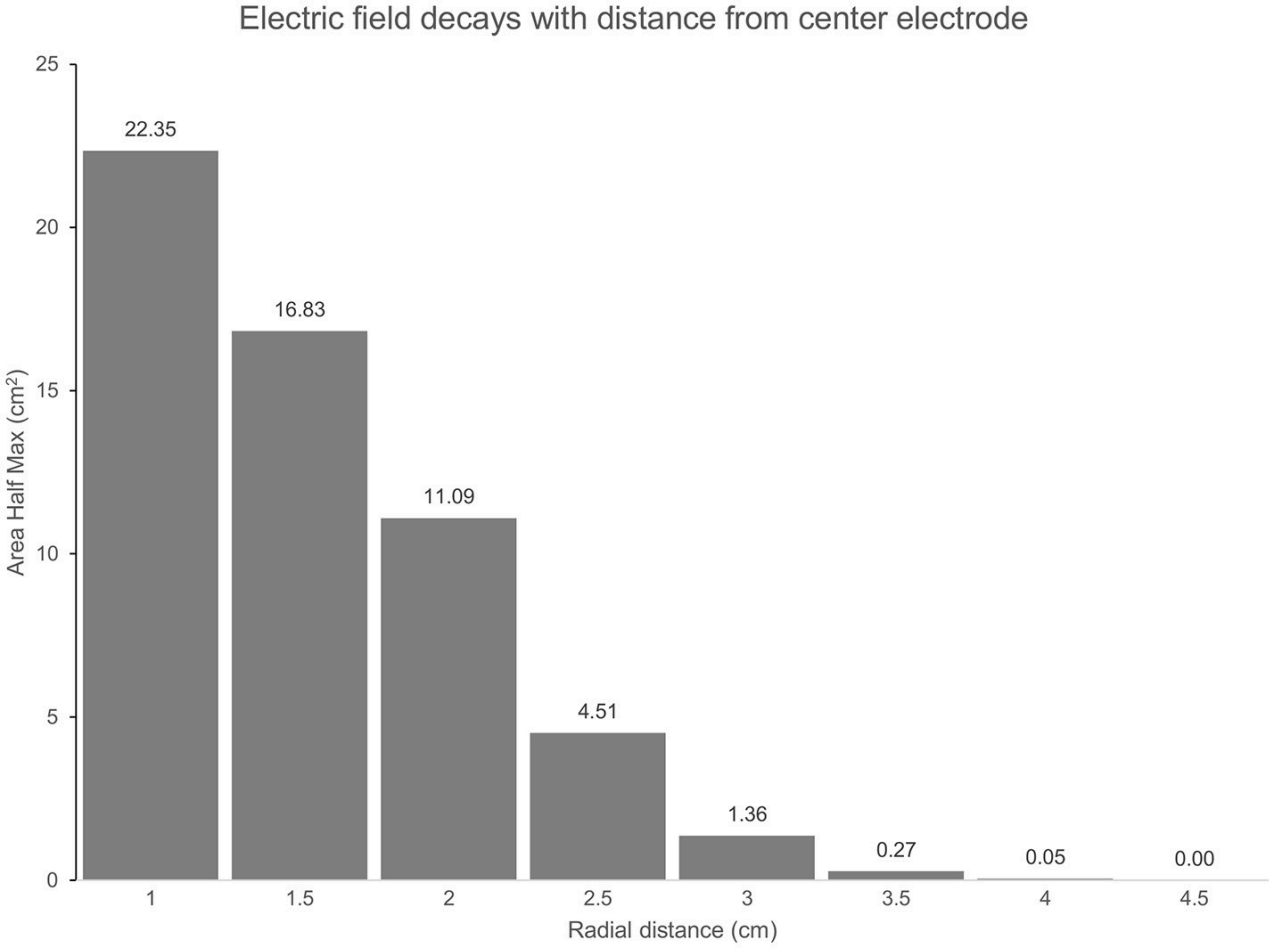
(TPJ Spread): The Area at Half Maximum (AHM) of the cortical electric field quantifies the spatial focality of the semicircular 3 × 1 HD-tDCS montage relative to the center electrode. In total, an area of 26 cm^2^ is above half the maximum E-field for each montage. As radial distance on the cortex below the center electrode increases, more of the AHM is excluded. At a 3 cm radius 5.23% (1.36 cm^2^) of the AHM remains, 0% is beyond 4.5 cm.

The predicted electric field from the semicircular 3 × 1 HD-tDCS montage produced a circular pattern as with previously modeled fully concentric 4 × 1 HD-tDCS electrode configurations (21). Peak electric field magnitude was situated underneath the central electrode for each stimulation intensity (Figure 2). The induced cortical electric field is mostly contained within the perimeter of the surrounding electrodes as quantified by the area of at least half maximum electric magnitude (26.05 cm^2^). The Area Half Max (AHM) is completely excluded outside the radius of the surrounding electrodes (5 cm); 0.18% (0.0469 cm^2^) of the AHM is contained outside 4 cm TPJ spread (Figure 3). Assuming a circular spatial distribution, the effective radius of the AHM can be calculated as *r* = (AHM/π)^0.5^, which results in an effective radius of 2.88 cm. While the relative spatial distribution is unchanged, field intensity decreases linearly with stimulation intensity from 0.65 V/m at 3 mA to 0.43 V/m at 2 mA to 0.22 V/m at 1 mA. The vestibular apparatus was more than 4.5 cm from the SMG (the ROI below the center electrode). As quantified in Figure 3, the Area Half Max (AHM) of the cortical electric field drops to zero. The electric field of the apparatus is also presented in Figure 2 as the ROI 'VR'. This restriction of stimulation intensity within the electrode array is consistent with principles of concentric HD stimulation previously studied (21).

### Safety and tolerability

The analyses of discomfort after each stimulation condition and the adverse effects after each stimulation session incorporated the data of the 3 experiments. Therefore, these analyses included 29 young-adult participants encompassing 87 sessions of HD-tDCS (29 sessions of each HD-tDCS condition: anode center, cathode center and sham), 783 blocks of 2 min of stimulation. The mean visual analog scale score of discomfort of each stimulation intensity and HD-tDCS condition are described in Table 1.

**Table 1:** Descriptive data of visual analog scale score for discomfort, with each HD-tDCS_3 × 1_ condition and current intensity.

	**SH Mean (SD) Median [IQ]**	**CC Mean (SD) Median [IQ]**	**AC Mean (SD) Median [IQ]**
Accommodation	–	4.4 ± 2.7	4.61 ± 2.1
		4.5 [2;7]	5 [3;6]
1 mA	0.66 ± 1.2	1.1 ± 1.7	1.0 ± 1.6
	0 [0; 1]	0 [0; 2]	0 [0; 1]
2 mA	1.8 ± 2.18	2.0 ± 1.9	2.2 ± 1.9
	1 [0; 3]	1 [1;3]	2 [1;4]
3 mA	2.8 ± 2.5	3.1 ± 2.5	3.2 ± 2.2
	2 [1;4]	3 [1;5]	3 [1;5]

*AC, anode center; CC, cathode center; SH, sham; SD, standard deviation; IQ, interquartile interval*.

There were 2 participants that reported mild to moderate headache after the sham stimulation sessions (one with the anode center condition and other with the cathode center condition). There were no further adverse effects reported. The frequency of the side effects observed in this study are described in Supplementary Material 2.

### Visual vertical trial

The visual vertical trial assessed 8 right-handed healthy subjects, mean age 26.4 ± 5.9 years, 5 women. Descriptive data of SVV are described in Table 2.

**Table 2:** Descriptive data of subjective visual vertical (SVV) for each HD-tDCS_3 × 1_ condition and current intensity.

**Current intensity**	**SH Mean (SD) Median [IQ]**	**CC Mean (SD) Median [IQ]**	**AC Mean (SD) Median [IQ]**
Baseline	0.19°± 0.96°	0.39°± 0.79°	0.00°± 0.09°
	0.38° [−0.26°; 0.96°]	0.30° [0.02°; 1.00°]	0.14° [−0.71°; 0.82°]
1 mA	−0.09°± 1.03°	−0.45°± 1.24°	−0.18°± 1.28°
	−0.02° [−0.83°; 0.54°]	−0.73° [−1.38°; 0.50°]	−0.20° [−0.97°; 0.86°]
2 mA	−0.01°± 1.13°	−0.84°± 1.28°	−0.05°±−1.29°
	0.07° [−0.82°; 0.76°]	−1.07° [−1.86°; 0.01°]	−0.05° [−0.93°; 0.98°]
3 mA	0.08°± 1.08°	−0.97°± 1.39°	0.05°± 1.22°
	0.11° [−0.63°; 0.84°]	−1.22° [−2.10°; −0.10°]	0.05° [−0.92°; 1.12°]

*AC, anode center; CC, cathode center; SH, sham; SD, standard deviation; IQ, interquartile interval. The results were expressed as degrees with positive values when the SVV tilt occurred in clockwise direction and negative values when SVV tilt occurred in counterclockwise direction*.

**Figure 4:**
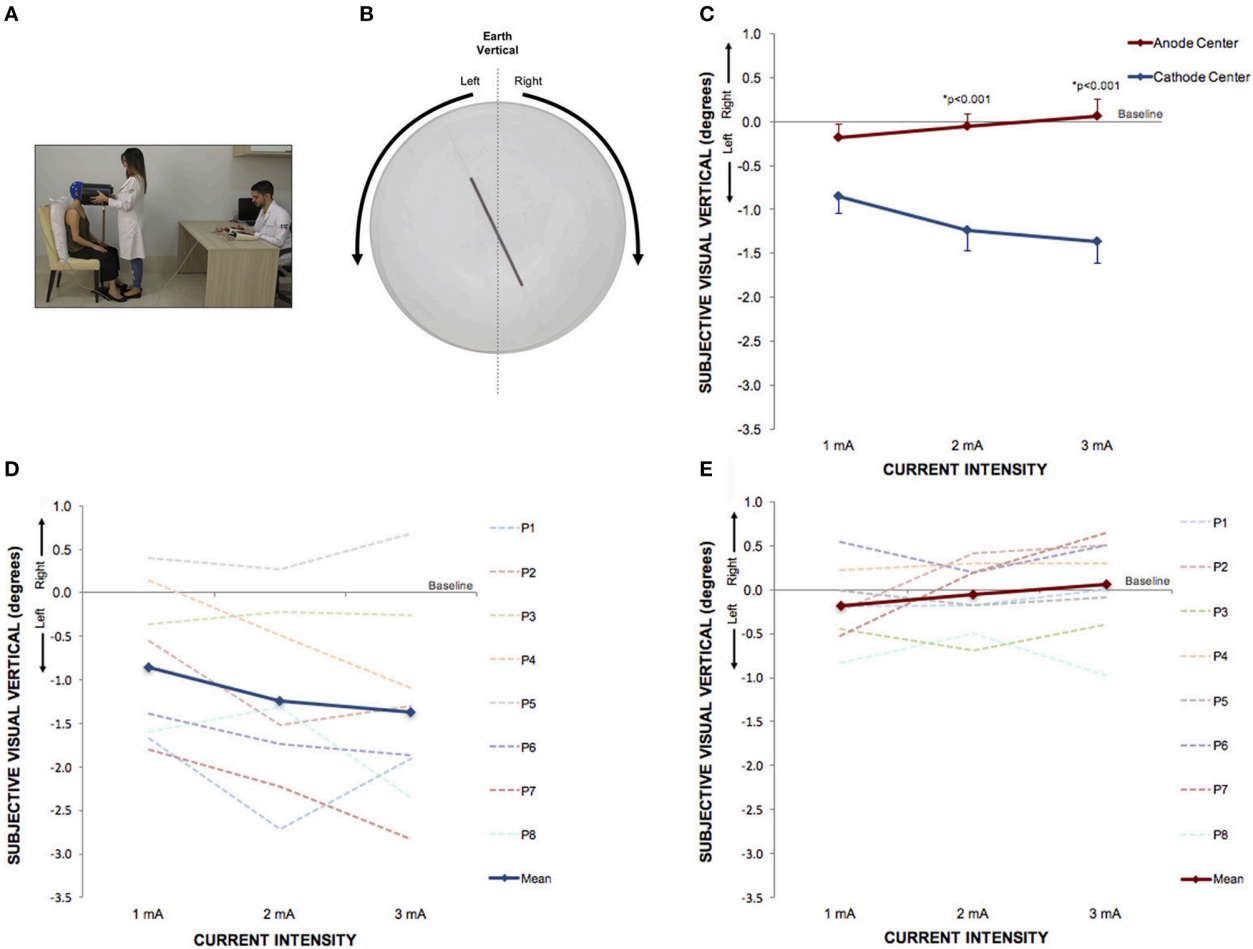
Perception of visual vertical can be manipulated by cathode center condition of HD-tDCS_3 × 1_ over the right temporo-parietal junction. **(A)** Experimental set up of the visual vertical trial. **(B)** The line inside the bucket used to assess visual vertical perception from the participant's perspective. The arrows illustrate the side of the bucket's rotation. **(C)** Difference between the SVV assessed at baseline and at current intensities of 1, 2, and 3 mA (mean; s.e.m.). There were intensity and polarity-dependent effects only after cathode center HD-tDCS_3 × 1_ condition. **(D)** Difference between the SVV assessed at baseline and at current intensities of 1, 2, and 3 mA after cathode center HD-tDCS_3 × 1_ condition of each participant showing the variability of the data and overall leftward tilt with progressively increase of SVV tilt with increasing stimulus intensity (mean; s.e.m.). **(E)** Difference between the SVV assessed at baseline and at current intensities of 1, 2, and 3 mA after anode center HD-tDCS_3 × 1_ condition of each participant showing the variability of the data and overall absence of effects. Written informed consent was obtained from the participants for the publication of this image.

Between each HD-tDCS_3 × 1_ condition at baseline, there was no significant difference in SVV. There were significant intensity dependent effects on the cathode center condition inducing leftwards SVV tilt (Kruskal–Wallis test: *p* = 2.52e-07; Tukey *post-hoc* tests comparisons: 0 and 1 mA: *p* = 0.004; 0 and 2 mA: *p* < 0.0001; 0 and 3 mA: *p* < 0.0001; 1 and 3 mA: *p* = 0.020). For assessment of stimulation polarity effects, the Kruskal–Wallis test revealed polarity difference for current intensity (1 mA: *p* = 0.0260; 2 mA: *p* < 0.0001; 3 mA: *p* < 0.0001). The Tukey *post-hoc* test showed a significant difference between the sham and cathode center condition at each current intensity (1 mA: *p* = 0.0263; 2 mA: *p* < 0.0001; 3 mA: *p* < 0.0001), and between sham and anode center after 2 mA (*p* = 0.0263), and between anode and cathode center in 2 mA (*p* < 0.0001) and 3 mA (*p* = 0.0001) (Supplementary Material 3). The results of SVV are illustrated in Figure 4.

### Postural control trial

The postural control trial studied 14 right-handed healthy subjects, mean age 24.5 ± 3.9 years, 8 women. Descriptive data of WBA are described in Table 3.

**Table 3:** Descriptive data of weight-bearing asymmetry (WBA) for each HD-tDCS_3 × 1_ condition and current intensity.

**Current Intensity**	**SH Mean (SD) Median [IQ]**	**CC Mean (SD) Median [IQ]**	**AC Mean (SD) Median [IQ]**
Baseline	−0.01 ± 6.99	0.47 ± 5.37	−0.16 ± 5.61
	0.48 [−1.01; 1.99]	1.78 [−2.75; 2.57]	0.03 [−2.59; 2.30]
1 mA	−0.44 ± 6.06	2.17 ± 6.03	−2.19 ± 5.01
	1.47 [−4.19 to 2.30]	2.04 [−0.28; 5.44]	−0.49 [−6.45; 2.03]
2 mA	−1.19 ± 6.77	1.55 ± 6.29	−0.55 ± 4.89
	−0.53 [−5.82; 2.06]	2.05 [−1.47; 4.88]	1.32 [−4.02; 2.25]
3 mA	−0.10 ± 7.33	1.92 ± 6.55	−2.39 ± 5.76
	1.20 [−4.62; 2.04]	2.26 [−0.19; 5.83]	−1.43 [−6.89; 2.33]

*AC, anode center; CC, cathode center; SH, sham; SD, standard deviation; IQ, interquartile interval. The results were expressed as the percentage of the total body weight loading with positive values when the WBA occurred toward the right side and negative values when WBA occurred toward the left side*.

**Figure 5:**
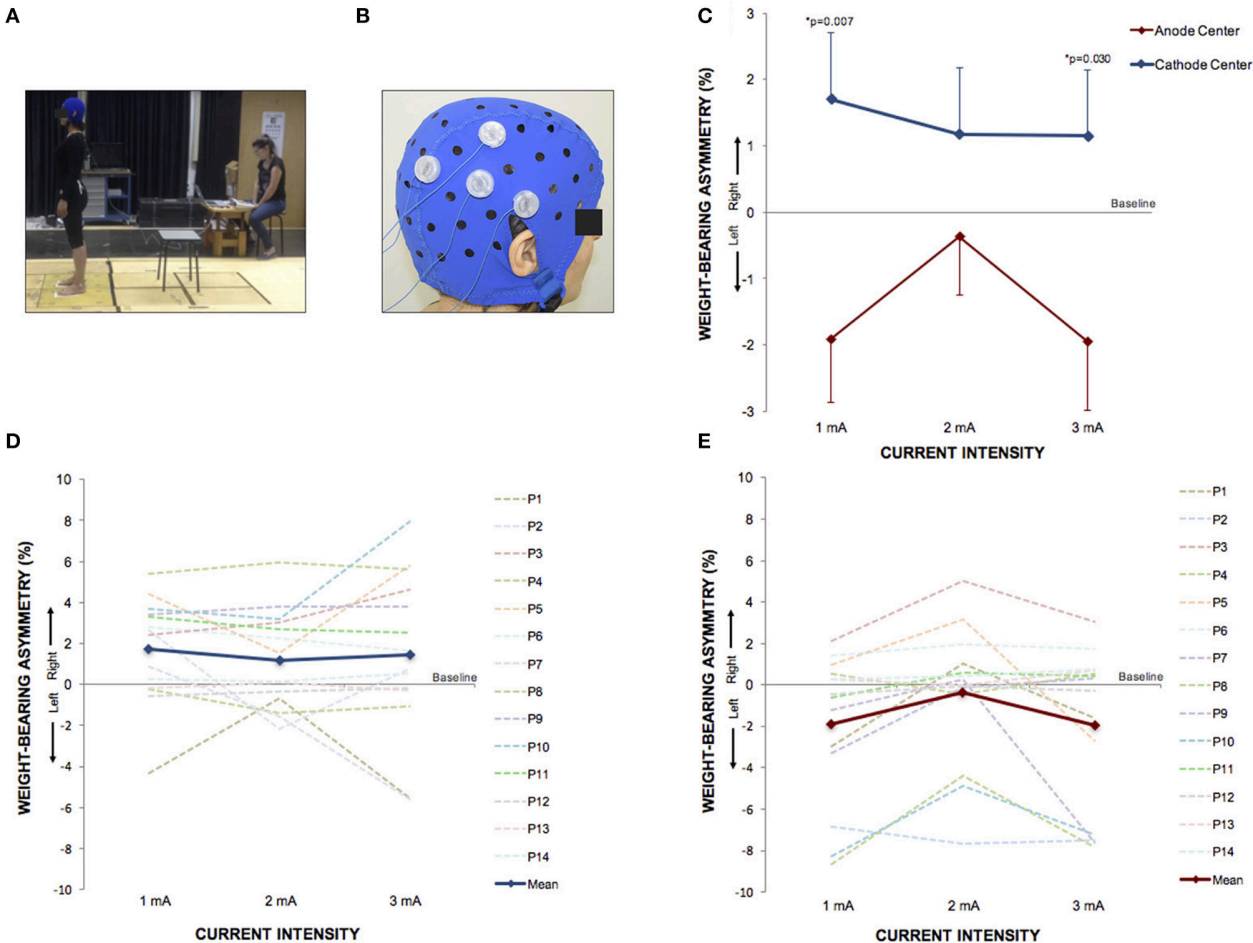
HD-tDCS_3 × 1_ over the right temporo-parietal junction can produce postural asymmetry. **(A)** Experimental set up of the postural control trial. **(B)** Position of the HD-tDCS_3 × 1_ electrodes in a female participant. **(C)** Difference between the WBA assessed at baseline and at current intensities of 1, 2, and 3 mA (mean; s.e.m). There were polarity-dependent effects that produced a rightward WBA after cathode center and leftward WBA after the anode center condition. **(D)** Difference between the WBA assessed at baseline and at current intensities of 1, 2, and 3 mA after cathode center HD-tDCS_3 × 1_ condition of each participant showing the variability of the data and overall effects toward the right side (mean; s.e.m.). **(E)** Difference between the WBA assessed at baseline and at current intensities of 1, 2, and 3 mA after anode center HD-tDCS_3 × 1_ condition of each participant showing the wide range of the data and overall effects toward the left side. Written informed consent was obtained from the participants for the publication of this image.

Between each HD-tDCS_3 × 1_ condition at baseline, there was no significant difference in WBA. The Kruskal–Wallis tests comparing WBA per current intensity among each stimulation condition resulted in no significant differences. However, the Kruskal–Wallis tests comparing polarity-dependent effects on WBA within all current intensities showed a significant difference (1 mA: *p* = 0.0080; 2 mA: *p* = 0.0221; 3 mA: *p* = 0.0323). The Tukey *post-hoc* test revealed that the differences occurred between sham and cathode center after 2 mA (*p* = 0.0215), and between cathode and anode center after 1 mA (*p* = 0.0068) and after 3 mA (0.0301) There was no difference between anode center and sham condition (Supplementary Material 4). The results of the WBA are illustrated in Figure 5. These results indicate that the cathode center condition presented more effect on WBA than anode center and sham conditions.

### EEG trial

The EEG trial included 7 right-handed healthy subjects, mean age 34.7 ± 7.6 years, 4 men. Descriptive data of channels 164 (EEG 10-20 CP4) and 66 (EEG 10-20 CP3) are presented in Table 4.

**Table 4:** Descriptive data of EEG gamma absolute power density of channels 164 (CP4) and 66 (CP3) for each HD-tDCS_3 × 1_ condition and current intensity.

**Current intensity**	**CHANNEL 164 (CP4) Median [IQR]**			**CHANNEL 66 (CP3) Median [IQR]**		
	**SH**	**CC**	**AC**	**SH**	**CC**	**AC**
Baseline	1.09	1.58	1.88	2.05	3.24	1.54
	[0.65; 1.64]	[1.07; 2.29]	[1.34; 4.56]	[1.53; 2.39]	[0.90; 4.35]	[1.43; 2.69]
1 mA	1.84	2.53	1.89	1.86	3.36	1.71
	[1.07; 2.53]	[1.28; 5.14]	[1.75; 2.61]	[1.16; 3.37]	[1.57; 13.33]	[0.94; 2.13]
2 mA	2.62	1.20	1.86	2.46	2.81	1.77
	[1.54; 3.19]	[0.90; 3.81]	[1.43; 3.44]	[1.71; 4.61]	[1.35; 10.23]	[1.32; 2.47]
3 mA	1.80	4.92	1.94	2.94	3.32	1.74
	[1.57; 4.866]	[1.36; 30.22]	[1.21; 2.54]	[1.69; 7.86]	[1.20; 59.07]	[1.25; 2.34]

*AC, anode center; CC, cathode center; SH, sham; IQ, interquartile interval*.

**Figure 6:**
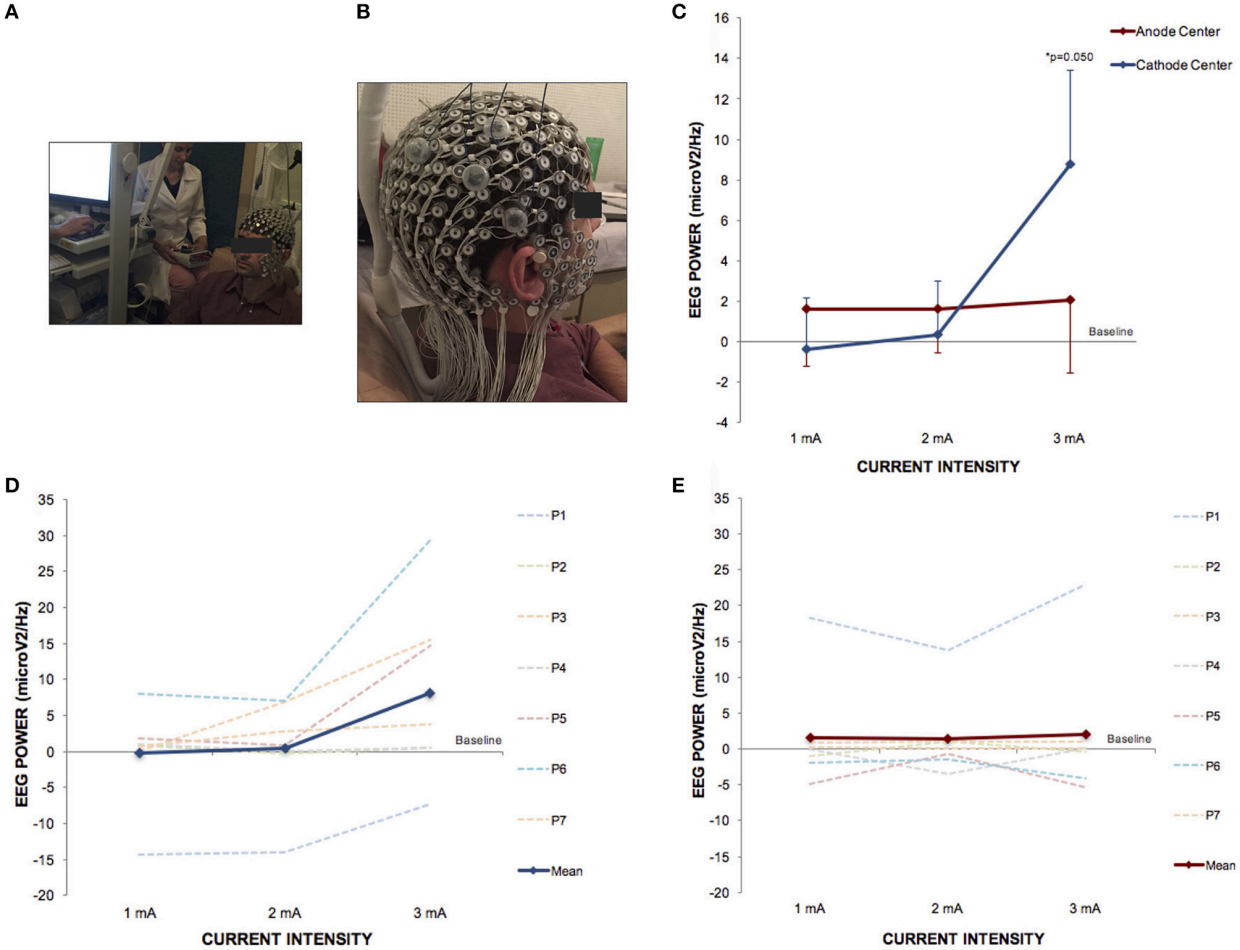
HD-tDCS_3 × 1_ over the right temporo-parietal junction can produce effects in power spectral density electroencephalography (EEG) of gamma frequency band. **(A)** Experimental set up of the EEG trial. **(B)** Position of the HD-tDCS_3 × 1_ electrodes in a male participant. **(C)** Difference between the EEG power assessed at baseline and at current intensities of 1, 2 and 3 mA (mean; s.e.m.). There was an increase of gamma power EEG after cathode center condition at current intensity of 3 mA. **(D)** Difference between the EEG gamma power assessed at baseline and at current intensities of 1, 2, and 3 mA after cathode center HD-tDCS_3 × 1_ condition of each participant showing the variability of the data and overall effects at current intensity of 3 mA. **(E)** Difference between the EEG gamma power assessed at baseline and at current intensities of 1, 2, and 3 mA after anode center HD-tDCS_3 × 1_ condition of each participant showing the variability of the data and overall no effects. Written informed consent was obtained from the participants for the publication of this image.

There was no significant difference in EEG-PSD between the HD-tDCS_3 × 1_ conditions at baseline in all frequency bands. There was a significant difference for polarity at a current intensity of 3 mA (Kruskal–Wallis test; *p* = 0.050) between cathode center and anode center conditions for EEG-PSD (Tukey *post-hoc* test; *p* = 0.050) on channel 164 in the gamma frequency band. The Kruskal–Wallis test revealed intensity-dependent effects on the cathode center condition (*p* = 0.046) in the gamma frequency band, and the Tukey *post-hoc* test indicated a significant difference between 2 and 3 mA (*p* = 0.044). The results of EEG-PSD of channel 164 are illustrated in Figure 6.

## Discussion

The present study provides evidence supporting manipulation of human *verticality* by targeting the right TPJ with high-definition transcranial direct current stimulation (using a 3 × 1 montage; HD-tDCS_3 × 1_). This represents an important advancement in understanding dose-response effects of tDCS, recently high-lighted by the US National Institutes of Health as a priority research area. As well, our experimental paradigm addresses a critical human function (verticality), implicated in health and disease (14–17). This is perhaps the first study using HD-tDCS to generate a “virtual lesion,” mimicking clinical symptoms at will (SVV direction-specific manipulation), having a host of applications in experimental neurology, and bringing in line with the more established technique of transcranial magnetic stimulation (29–31), a powerful experimental tool over the last two decades. In the field of human balance (upright stance and gait), the prior art is galvanic vestibular stimulation, strongly manipulating verticality, dynamic stance and gait, in a polarity-dependent manner, thought to be caused by direct stimulation of the vestibular apparatus (32, 33). Here we show modification of multimodal cortex, sparing peripheral vestibular stimulation, yet potentially with comparable end effects.

We present a novel approach to systematically analyze the dose-response effects of a focal HD-tDCS stimulation montage, using behavioral and physiological outcome measures. This repeated-measures design enabled comparison of 3 different current intensities (1, 2, and 3 mA) using anode center, or cathode center, relative to a sham stimulation condition. We centered the montage over the right TPJ in healthy subjects, based on prior data that we could manipulate perception of verticality (18), which is of major clinical importance in adult falls and post-stroke recovery (14–17), and thus has potential clinical utility. Furthermore, many prior tDCS studies have relied on manipulation of the MEP when targeting primary motor cortex to examine tDCS effects (34), and here we demonstrate behavioral and physiological effects in multimodal cortex. We showed that HD-tDCS can be applied repeatedly in a safe and tolerable manner.

To our knowledge we are the first to apply HD-tDCS using currents of 3 mA. With no reports of adverse effects (note: sham condition, headache was reported), this provides evidence of good tolerability, and is likely because the total current dose for this protocol was under the recommended safety limits (2). We also attribute the tolerability to the accommodation protocol. The use of pre-treatment mild topical anesthetic containing a low-concentration of benzocaine has been previously described to reduce subjective discomfort (35). This is the first time that a tDCS accommodation protocol is suggested to induce gradual desensitization and, therefore, increase tolerability of participants. The advantages of using a non-chemical strategy to reduce the discomfort are the absence of adverse events from a chemical anesthetic (which can mask pain from potential skin burn), and the gradual accommodation of subjects regarding the current that may avoid a psychophysical reaction to this type of intervention.

To-date, there are only two studies reporting dose-response effects of HD-tDCS in human adults (36, 37). The crossover trial of Shekhawat et al. investigated 1 and 2 mA applied for 10 and 20 min, applied over the left temporo-parietal area or the dorsolateral prefrontal region in patients with tinnitus; describing positive effects of both intensities and most effective tinnitus relief after 2 mA for 20 min (36). In contrast, Castillo-Saavedra et al. aimed to establish the number of HD-tDCS sessions to achieve a reduction in pain from fibromyalgia, and indicated that a median number of 15 sessions would be recommended to achieve clinically meaningful outcomes (37). The present crossover clinical trails compared the effects of 3 repetitions of 1, 2, and 3 mA, each for only 2 min and found intensity-dependent after-effects on SVV and EEG. Although there are distinctions between the studies described above, a common feature is the indication that higher doses of HD-tDCS_3 × 1_ may produce stronger effects. Previous investigations have indicated that larger lesion volume after stroke may induce greater visual vertical tilt (10). The results from the modeling of the HD-tDCS_3 × 1_ induced current flow indicates that higher current-intensities result in greater brain area penetration, which may explain our linear intensity-dependent effects on visual vertical perception.

The challenges of analyzing dose-response effects of 3 different levels of transcranial stimulation on clinical outcomes are different from experiments that analyzed only physiological outcomes such as cortical excitability (38). To achieve clinical effects, the intervention may not only produce local changes of cortical excitability but also neural network neuromodulation strong enough to change perception or behavior (39, 40). Still, changing behavior might require more complex neural network plasticity than changing one aspect of perception or one neurological feature. We found polarity-dependent clinical effects on visual vertical perception and also on postural control. The cathode center HD-tDCS_3 × 1_ condition induced tilting effects of SVV away from the side of stimulation and asymmetric load toward the side of stimulation. It was described that most patients after stroke present with visual vertical deviation away from the hemispheric lesion side and asymmetric load toward the ipsilesional leg (10, 22, 41). Therefore, the polarity effects of visual vertical perception and postural control WBA induced in healthy subjects by our protocol have replicated the effects of encephalic lesions after stroke (10, 22, 41). Baier et al. indicated that the superior temporal gyrus plays a role exclusively after right-sided lesions and ipsilesional SVV tilt might follow more complex lesions of the neural network that process verticality perception after stroke (10). Therefore, considering that our right TPJ target (which involves the superior temporal gyrus) was functionally uniform under our protocol, the homogeneous polarity effects of the cathode center HD-tDCS_3 × 1_ condition on visual vertical perception observed were physiologically reasonable. The less prominent effects on postural control in relation to vertical perception observed in the pilot results presented here was expected since the postural control represents a multimodal task involving several complex neural networks, including vertical perception (17, 22).

Recently, we induced temporary SVV misperception in healthy subjects in a direction away from the cathodal stimulation side using conventional bipolar bilateral tDCS applied over the temporo-parietal region (18). Here we also induced a SVV misperception in healthy subjects toward the same direction using cathode center HD-tDCS_3 × 1_ over the right TPJ. Thus, the present results strengthen our previous hypothesis that the underlying mechanism of the polarized effects of tDCS over TPJ is related to an inter-hemispheric misbalance of neuronal excitability within the verticality perception network. We speculate that cathode center HD-tDCS_3 × 1_ condition over the left hemisphere would induce SVV tilt toward the right side since we observed this effect by using conventional tDCS with cathodal electrode over the left hemisphere. However, the effects of the HD-tDCS_3 × 1_ over the left TPJ might be less prominent than over the right TPJ due to the dominance of verticality perception in the right hemisphere (42). Further studies will be necessary to evaluate the effects of HD-tDCS_3 × 1_ over the left TPJ on verticality perception and postural control.

The polarity effects of the HD-tDCS applied over TPJ indicate that the stimulation produced a top-down influence and high-level process of modulation. The nearest electrode to the peripheral vestibular system of our 3 × 1 montage of HD-tDCS is the surrounding electrode over temporal lobe (Figure 1). In the cathode center condition, the HD-tDCS surround electrodes are classified as anode electrodes (7). If our stimulation had produced a galvanic vestibular stimulation, the expectation would be SVV tilt toward the right side (toward the side of the anode) (43). Recently, Volkening et al. have observed tilt toward the anode during GVS and mild tilt toward the cathode after 20 min of stimulation (33). Our modeling of the HD-tDCS current flow included, for the first time, the peripheral vestibular system and showed negligible current flow under these vestibular structures.

Although the dense-array EEG (HD-EEG) has been widely used, it is seldom described with HD-tDCS imbedded. An advantage of HD-EEG is the temporal resolution associated with source localization obtained by the coregistration of the 256 channels data with neuroanatomical MRI (44). The feasibility of this innovative method was proved in the present study.

Our EEG results showed intensity-dependent effects of HD-tDCS_3 × 1_ observed by an increase in gamma oscillations (Figure 5). There is previous evidence indicating that the gamma oscillations of the TPJ might be a neural signature of visual perception (45). Beauchamp et al. have hypothesized that if TPJ gamma oscillations are critical for visual perception, then disrupting them would be expected to interfere with perception (45). Also, gamma oscillations in right hemisphere parietal areas are associated with visuo-motor tasks (46).

Our translational study results have indicated that the same protocol of HD-tDCS_3 × 1_ over TPJ can influence gamma oscillations, visual misperception, and postural motor control. Using our HD-tDCS_3 × 1_ montage and protocol for the three clinical trials, we showed behavioral and physiological effects specific to polarity and dose. Our protocol was feasible, safe and well tolerated, with an accommodation protocol shown to be helpful in decreasing stimulation discomfort, without chemical use. Our results provide credible evidence for HD-tDCS_3 × 1_ to have focal effects on physiology, associated with behavioral change, and critical dependence on stimulation polarity and intensity. These findings support the use of HD-tDCS_3 × 1_ as a focal method of neuromodulation, and warrants further studies in clinical populations with this protocol.

In the present study, we reasoned form prior published studies, that although applied to a relatively novel brain target using behaviorally-specific outcome measures that are non-standard in the tDCS field, we may logically see effects that are dependent on stimulation polarity; consistently shown in the literature in studies of the human motor system. It is also reasonable to predict that current intensity (that ultimately affects charge density at the cortical level), will, within limits, produce a greater effect when the current is stronger than weak. Our finding support this, yet we do not maintain that this relationship is a rule, and we anticipate that further increases in dose (intensity or montage or duration) may not, and likely not yield ever increasing effects; that intensity and polarity effects in our measured domain may not transfer to other outcome measures, and finally, that our findings in TPJ, may not extend to other brain regions.

## Ethics statement

This study was conducted according to the Helsinki Declaration requirements for human investigation and was approved by the Ethics Committee for Analysis of Research Projects, Hospital das Clinicas de Ribeirao Preto, University of Sao Paulo (CAAE: 09485212.7.0000.5440). All participants provided written informed consent.

## Author contributions

TS: study concept and design of the three clinical trials [SVV, WBA, and electroencephalography (EEG)], interpretation of computational modeling, data acquisition, analysis and interpretation of the three clinical trials, and manuscript writing. DF: data acquisition, analysis and interpretation of the three clinical trials (SVV, WBA, and EEG), and critical revision of the manuscript for intellectual content. IT: optimization of the EEG protocol, EEG data acquisition, analysis and interpretation, and critical revision of the manuscript for intellectual content. DN: statistical analysis and interpretation of data of the three clinical trials (SVV, WBA, and EEG), and critical revision of the manuscript for intellectual content. BR: acquisition, analysis and interpretation of data of the SVV clinical trial, and critical revision of the manuscript for intellectual content. EB: data acquisition, analysis and interpretation of the WBA clinical trial, and critical revision of the manuscript for intellectual content. TL: study concept and design, data pre-processing, analysis and interpretation of the WBA clinical trial, and critical revision of the manuscript for intellectual content. DT: study concept and design, conduction and interpretation of computational modeling, and critical revision of the manuscript for intellectual content. AD: supervising optimization and data mining support. BM: EEG data preprocessing procedures. RM: data supervision and interpretation of the WBA clinical trial, and critical revision of the manuscript for intellectual content. FL: supervision of the statistical analysis and interpretation of data of the three clinical trials (SVV, WBA, and EEG), and critical revision of the manuscript for intellectual content. MB: study concept and design of the three clinical trials (SVV, WBA, and EEG), supervision and interpretation of computational modeling, and critical revision of the manuscript for intellectual content. JL: study concept and design of the three clinical trials (SVV, WBA, and EEG), data supervision and interpretation of the EEG clinical trial, and critical revision of the manuscript for intellectual content. DE: study concept and design of the 3 clinical trials (SVV, WBA, and EEG), interpretation of computational modeling, data analysis and interpretation of the three clinical trials and critical revision of the manuscript for intellectual content.

### Conflict of interest statement

CUNY has patents on brain stimulation with MB as inventor. MB has equity in Soterix Medical. The remaining authors declare that the research was conducted in the absence of any commercial or financial relationships that could be construed as a potential conflict of interest. The reviewer CB and handling Editor declared their shared affiliation.
