# Supplementary material for: Manipulation of Human Verticality Using High-Definition Transcranial Direct Current Stimulation
Source: Front Neurol. 2018 Oct 22;9:825. doi: 10.3389/fneur.2018.00825 (PMC6232937; doi:10.3389/fneur.2018.00825)
Supplement: Supplementary file 1 [file Data_Sheet_1.DOCX]

Manipulation of human verticality using high-definition transcranial direct current stimulation

**SUPPLEMENTARY MATERIAL**

**Supplementary material 1:**

**
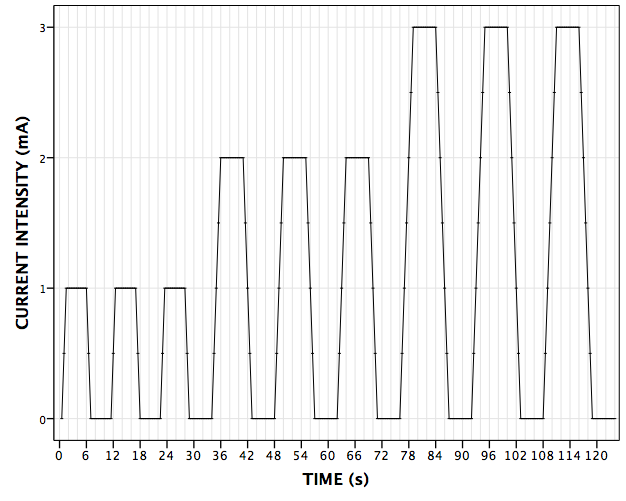
**

**Supplementary Fig. 1** Illustration of the accommodation stimulation protocol. This accommodation protocol was used to induce gradual desensitization and, therefore, increase tolerability for participants. It was composed of 3 repetitions of 5 seconds of stimulation at current intensities 1, 2 and 3 mA, with ramp-up and ramp-down of 2 s/mA and intervals of 5 seconds.

**Supplementary material 2:** Safety profiles at each HD-tDCS_3x1_ condition.

| **Adverse Events** | **SH** | **CC** | **AC** | |
| --- | --- | --- | --- | --- |
| **Seizure (n)** | 0 | 0 | | 0 |
| **Headache (n)** | 2 | 0 | | 0 |
| **Scalp pain (n)** | 0 | 0 | | 0 |
| **Neck pain (n)** | 0 | 0 | | 0 |
| **Itching (n)** | 0 | 0 | | 0 |
| **Tingling (n)** | 0 | 0 | | 0 |
| **Burning (n)** | 0 | 0 | | 0 |
| **Sleepiness (n)** | 0 | 0 | | 0 |
| **Trouble concentrating (n)** | 0 | 0 | | 0 |
| **Mood change (n)** | 0 | 0 | | 0 |

AC: anode center; CC: cathode center; SH: sham; n=number. The findings highlight tolerability and safety, despite unprecedented stimulation intensity using HD-tDCS, with the only adverse effects apparent with sham stimulation.

**Supplementary material 3:** Results of Kruskal-Wallis and Tukey post-hoc tests comparing stimulation conditions among each current intensity of subjective visual vertical (SVV).

| **Current intensity** | **Kruskal-Wallis test**  **(p-value)** | **Tukey Post-hoc Test**  **(p-value)** | | |
| --- | --- | --- | --- | --- |
|  |  | **SH-AC** | **SH-CC** | **AC-CC** |
| **1 mA** | **0.0260*** | 0.8132 | **0.0263*** | 0.1191 |
| **2 mA** | **<0.0001*** | **<0.0001*** | **<0.0001*** | **<0.0001*** |
| **3 mA** | **<0.0001*** | 0.9999 | **<0.0001*** | **<0.0001*** |

AC: anode center; CC: cathode center; SH: sham.

**Supplementary material 4:** Results of Kruskal-Wallis and Tukey post-hoc tests comparing stimulation conditions among each current intensity of weight-bearing asymmetry (WBA).

| **Current intensity** | **Kruskal-Wallis test**  **(p-value)** | **Tukey Post-hoc Test**  **(p-value)** | | |
| --- | --- | --- | --- | --- |
|  |  | **SH-AC** | **SH-CC** | **AC-CC** |
| **1 mA** | **0.0080*** | 0.6146 | 0.0914 | **0.0068*** |
| **2 mA** | **0.0221*** | 0.7741 | **0.0215*** | 0.1183 |
| **3 mA** | **0.0323*** | 0.7583 | 0.1603 | **0.0301*** |

AC: anode center; CC: cathode center; SH: sham.
